# Reliability and validity of the Chinese version of the Athens insomnia scale for non-clinical application in Chinese athletes

**DOI:** 10.3389/fpsyg.2023.1183919

**Published:** 2023-09-15

**Authors:** Chenhao Tan, Jinhao Wang, Guohuan Cao, Chao Chen, Jun Yin, Jiaojiao Lu, Jun Qiu

**Affiliations:** ^1^Shanghai Research Institute of Sports Science (Shanghai Anti-Doping Agency), Shanghai, China; ^2^No.1 High School Affiliated to Tongji University, Shanghai, China

**Keywords:** AIS-NCA, athlete, non-clinical, sleep problem, daytime functioning

## Abstract

**Purpose:**

This study aimed to revise and examine the reliability and validity of the Chinese version of the Athens Insomnia Scale for Non-clinical Application (AIS-NCA) among Chinese athletes. Additionally, the study tested the scale in non-athlete individuals with similar sleep management practices to further analyze its cultural specificity among Chinese athletes and make preliminary inferences about its applicability in other Chinese populations.

**Methods:**

Four hundred twenty-six Chinese professional athletes and 779 high school students participated in this research. Both athletes and students were divided into two parallel groups for exploratory and confirmatory factor analyses. Additionally, three athlete samples and one student sample were established for reliability and validity assessments. Among athletes, the Pittsburgh Sleep Quality Index, the Epworth Sleepiness Scale, the Athlete Sleep Screening Questionnaire, and the Warwick-Edinburgh Mental Well-Being Scale were employed to evaluate convergent and discriminant validity. Re-test reliability was evaluated at intervals of 1 and 2 weeks. In the case of students, convergent and discriminant validity were tested using the Pittsburgh Sleep Quality Index and the General Self-Efficacy Scale, with re-test reliability assessed at two-week intervals.

**Results:**

The Chinese version of the AIS-NCA consists of six items, categorized into two dimensions: sleep problems and daytime functioning. This structure explained 65.08% (athletes) and 66.22% (students) of the variance. Confirmatory factor analysis revealed good model fit, with values of χ^2^/*df* = 2.217, CFI = 0.975, AGFI = 0.929, TLI = 0.953, and RMSEA = 0.076 among athletes, and χ^2^/*df* = 3.037, CFI = 0.979, AGFI = 0.947, TLI = 0.961, and RMSEA = 0.072 among students. The scale demonstrated a reasonable degree of measurement invariance. The overall scale and two subscales exhibited strong reliability and validity among athletes. Similar results in terms of reliability and validity were also observed within the student sample.

**Conclusion:**

The Chinese version of the AIS-NCA shows promise as an assessment tool for evaluating the sleep quality of Chinese athletes. It effectively captures both sleep-related concerns and daytime functionality within the athlete population. The scale demonstrates solid reliability and validity in professional athletes and holds potential for application across various other demographic groups in China.

## Introduction

Sleep is widely acknowledged as a fundamental component of human health (Buysse, 2014). Adequate sleep is essential for individuals to accomplish personal and professional responsibilities effectively. This is particularly crucial for athletes, as they rely on sleep as a critical method for fatigue recovery (Halson, 2013; Malhotra, 2017; Bonnar et al., 2018). Research has demonstrated that sleep quality is linked with cognitive development, recovery from cognitive impairment or fatigue, and injury recovery for athletes (Chennaoui et al., 2021; Leong and Chee, 2022; Cook and Charest, 2023). Conversely, poor sleep quality or sleep deprivation hinders recovery from fatigue and impairs athletic performance, increasing susceptibility to sports injuries (Gupta et al., 2017; Dwivedi et al., 2019; Charest and Grandner, 2022; Cook and Charest, 2023). Athletes and coaches have recognized the importance of sleep in enhancing athletic performance and have integrated it into their training and event planning (Venter, 2014; Walsh et al., 2021).

Research investigating sleep quality in professional athletes has indicated suboptimal sleep quality in this population (Gupta et al., 2017; Kölling et al., 2019). Athletes’ sleep quality may be inferior to that of the general population due to stress, training fatigue, or other factors related to training and competition (Lastella et al., 2018; Fullagar et al., 2019; Lastella et al., 2020; Vitale et al., 2022; Halson et al., 2022a,b). These observations, along with the need for enhanced athletic performance, have spurred research aimed at analyzing and intervening to improve athletes’ sleep quality characteristics (Bonnar et al., 2018; Dunican et al., 2019; Gwyther et al., 2022; Vitale et al., 2022). Consequently, the assessment of sleep quality among athletes and the implementation of interventions have become critical topics in the field of sports science.

In sports science research, the measurement of sleep quality is crucial when analyzing athletes’ sleep patterns and implementing interventions (Halson, 2019). Scales are the most widely used approach to evaluate sleep quality in research. Studies examining athletes’ sleep quality, identifying factors influencing sleep quality, and implementing interventions, such as Cranial Electrotherapy Stimulation and sleep hygiene, have primarily relied on scales, such as the Pittsburgh Sleep Quality Index (Van Ryswyk et al., 2017; Kwon et al., 2019; Driller et al., 2022; Suppiah et al., 2022; Monma et al., 2023). While these scales have facilitated significant advancements in sleep quality research among athletes, their limitations and potential biases should not be overlooked.

Specifically, most scales face the critical question that they were originally designed to aid in clinical diagnosis or to meet other clinical needs for sleep measurement (Buysse, 2014; Mollayeva et al., 2016; Sattler et al., 2021). The question of whether sleep scales developed for clinical diagnosis or other clinical needs are applicable to athletes who do not experience insomnia or have suboptimal sleep quality remains unresolved. Furthermore, it is noteworthy that the positive aspects of sleep are increasingly gaining attention (Buysse, 2014). The traditional focus of sleep medicine on sleep disorders and deprivation has proven inadequate, and the mere absence of disease is no longer considered sufficient to meet the present-day demands on sleep (Buysse, 2014). Therefore, it is uncertain whether the use of clinical sleep scales can effectively evaluate sleep quality among athletes in non-clinical settings, and their ability to provide reliable insights into athletes’ sleep beyond clinical considerations is questionable.

Several studies have revealed the limitations of sleep scales, which are commonly used in sports science research and practice. For instance, Driller and colleagues utilized the Pittsburgh Sleep Quality Index to compare sleep differences among athletes in different sports but found no differences in the overall PSQI score (Driller et al., 2022). However, when the scores of some of the questions were analyzed, they revealed differences between sports. The breakdown of PSQI components to describe sleep is common in such studies (Gupta et al., 2017; Bender et al., 2018a,b; Driller et al., 2022; Romdhani et al., 2022; Halson et al., 2022a,b). It may serve as a compromise between the scale and the population. Additionally, a review of studies on sleep quality problems in athletes found that the PSQI may not capture some sleep issues that occur in certain cases (Gupta et al., 2017), raising doubts about the validity of clinical scales for non-clinical patient studies.

To address this gap, Sattler and colleagues have developed a sleep scale for non-clinical use, called the Athens Insomnia Scale for Non-Clinical Application (AIS-NCA; Sattler et al., 2021). They noted that most sleep questionnaires used in current research are primarily intended for clinical use, screening populations, and then identifying individuals with sleep disorders for diagnosis or intervention. While these measurement tools are essential for clinical purposes and can help with diagnosis and intervention, there is a greater need in practice for a scale that can more precisely reflect the severity of sleep problems, daytime functioning, and other relevant factors. Such a scale would be helpful in studies with a preventive purpose. Additionally, the scale should be short and concise and adaptable to specific populations or limited measurement time, depending on the study’s needs, particularly in studies that require a large number of scales to be completed within a limited time to analyze factors that could play a preventive role.

AIS-NCA arose from this background and need (Sattler et al., 2021). The scale was adapted from the Athens Insomnia Scale (AIS), which has been shown to be a useful substitute for the Pittsburgh Sleep Quality Index (Kawaratani et al., 2022). Due to its excellent quality, the AIS was chosen as the starting point for developing a sleep scale suitable for non-clinical settings.

Specifically, the AIS-NCA introduced several modifications to the original AIS: (1) Some items were rephrased to increase the versatility. (2) The 4-point scale was replaced with a 5-point scale, enhancing measurement precision and facilitating the identification of differences in sleep quality among non-clinical populations. (3) The response options for each item were rewritten to balance positive and negative states, making them better suited for describing sleep characteristics in non-clinical populations. As a result, the AIS-NCA consists of seven items, each scored on a five-point scale, with all items categorized into two dimensions: sleep problems and daytime function. For a timeframe of 1 year and 1 month, the AIS-NCA demonstrated good reliability and validity (Sattler et al., 2021). Since its release, researchers have employed the scale in studies of medical staff during the COVID-19 pandemic (Reicherts et al., 2022), offering a constructive means to evaluate sleep quality in these workers. Moreover, the scale has been utilized in large-scale investigations concerning the effects of risk exposure and prognosis on sleep during the pandemic, leading to the proposal of recommendations for improving sleep quality based on the findings (Zerbini et al., 2022), thereby providing scientific support for public health promotion strategies during this challenging time.

It should be emphasized that the AIS-NCA is not intended to replace existing tools but extends their utility for non-clinical purposes. The AIS, being an excellent insomnia screening tool, has been employed in both clinical and non-clinical individuals studies, demonstrating high diagnostic validity in differentiating between the two groups (Soldatos et al., 2003; Chiang et al., 2009; Chung et al., 2011). However, these studies used the AIS primarily as a screening tool for identifying insomnia disorder, as it was initially designed (Soldatos et al., 2003). For non-clinical people, particularly groups like athletes who prioritize sleep health and its impact on athletic performance, the focus is not solely on identifying sleep disorders but rather on measuring and describing changes in sleep quality itself. Assessing the extent of positive or negative sleep quality is crucial for them, rather than exclusively concentrating on more extreme negative situations. This approach aligns with the emphasis on evaluating sleep quality in the context of sleep health (Buysse, 2014). The enhanced generalizability and balanced evaluation in AIS-NCA are intended to complement the AIS, catering to this specific need.

A similar situation is observed in the realm of sleep measurement tools for athletes. The Athlete Sleep Screening Questionnaire (ASSQ) is the sole available screening tool explicitly designed to assess athletes’ sleep quality (Samuels et al., 2016). The primary purpose of this questionnaire is to determine the need for further screening, diagnosis, or intervention among athletes, focusing on screening and intervention recommendations (Samuels et al., 2016; Gouttebarge et al., 2020). The sleep difficulties subscale within the ASSQ holds particular significance, as users rely on critical thresholds to identify athletes with potential sleep quality issues, warranting intervention. However, the emphasis on screening for sleep problems restricts the broader application of the ASSQ in daily training assessments and research involving athletes, especially in the context of sleep quality as a novel approach to enhancing athletic performance. This further emphasizes the necessity of applying AIS-NCA to the athlete population.

In summary, AIS-NCA offers a new perspective and methodological supplement for assessing athletes’ sleep quality. Although the Athlete Insomnia Screening Scale (AIS-NCA) has been studied for public health or disease prevention purposes and has shown good validity and practical value (Sattler et al., 2021; Reicherts et al., 2022; Zerbini et al., 2022), there is still a lack of research on its use in athletes. In recent years, researchers have recommended the use of scales that are specifically designed for athletes to evaluate sleep and account for some of the unique characteristics of athletes (Samuels et al., 2016; Rabin et al., 2020). Therefore, before using the AIS-NCA in the athlete population for non-clinical purposes, it is necessary to revise and test the scale’s validity and reliability in athletes.

Moreover, it is crucial to highlight that the AIS-NCA functions as a self-report scale. Sleep quality lacks a universally agreed-upon definition (Harvey et al., 2008), implying that individual perceptions of sleep inherently shape its structure, particularly within self-report scales. This suggests that the structure of the AIS-NCA among Chinese athletes may be influenced by two main factors: Chinese culture and the athletic profession. Regarding the former, previous research using the widely utilized Pittsburgh Sleep Quality Index (PSQI) has already unveiled slight variations in its structure across different countries, even among similar university student populations (Gelaye et al., 2014). Chinese cultural influences on Chinese athletes could manifest in specific characteristics within the scale’s structure. As for the latter, studies investigating the structure of the PSQI among diverse populations have identified considerable variability (Manzar et al., 2018), including among Chinese student populations, where distinct structures have been observed among adolescents and university students (Guo et al., 2016; Guo, 2022). This implies that the AIS-NCA’s structure and validity characteristics among Chinese athletes may also be associated with their athletic profession. Therefore, examining the potential influences of cultural and occupational/population factors is imperative during revising and testing the AIS-NCA among Chinese athletes. While this examination will not impact the applicability of AIS-NCA among athletes, such expansion holds significant promise for future cross-group and cross-cultural comparative research. Additionally, it can offer preliminary insights for applying AIS-NCA among other Chinese populations, thus laying the groundwork for methodological approaches in future cross-group and cross-cultural comparative studies.

This study aimed to revise the Chinese version of AIS-NCA among Chinese athletes and evaluate its reliability and validity. The reference frame was set to 1 month (4 weeks), as was done in a previous study (Sattler et al., 2021), and was decided based on the practical requirements and feasibility of using the AIS-NCA in athletes.

Moreover, to consider potential cultural or professional variations in the perception and attitudes toward sleep, this study conducted supplementary tests with a sample of Chinese students whose daily routines and time management resembled those of athletes. The aim was to preliminarily distinguish whether there are distinctive patterns in the structure linked to mainland Chinese culture or the athlete population. The daily routine management of Chinese athletes resembles that of students, with the primary distinction being that athletes prioritize sports training over academic courses as their primary daily task. Employing students as a control group enables the examination and control of factors associated with daily routine management and the influence of social jet lag, thereby circumventing the introduction of additional variations due to disparate sleep patterns and emphasizing the principal occupational characteristic of professional sports training. If the structure obtained from the athletes deviates from the original scale but aligns with that of the student sample, it may imply the impact of cultural factors. Conversely, significant disparities between the athlete and student samples would indicate the influence of the athletic profession itself.

## Materials and methods

### Study design

This is a descriptive, cross-sectional, and methodological study. The initial step encompassed the translation of AIS-NCA into Chinese. This translation process for AIS-NCA involved five stages: forward translation, meticulous evaluation by professionals in psychology and sports science, pilot testing and meticulous refinement of phrasing, subsequent back translation, and a series of iterative revisions guided by semantic considerations, culminating in the establishment of a consensus for the Chinese version.

Subsequent to obtaining the Chinese version of AIS-NCA, this study is prominently dedicated to exploring the structure of the Chinese rendition and validating its psychometric properties through cross-sectional surveys. In this phase, the AIS-NCA was subjected to thorough analysis and examination within two distinct cohorts: Chinese professional athletes and students whose daily routines closely paralleled those of Chinese athletes.

### Participants

Four hundred twenty-six professional athletes and 779 high school students voluntarily participated in this study.

The professional athletes were recruited from Shanghai-based professional sports teams, and the students were recruited from a senior high school in Shanghai. The athletes were randomly divided into two parallel samples according to their gender and sports categories (including fencing, modern pentathlon, badminton, table tennis, shooting, archery, boxing, judo, basketball, softball, hockey, handball, Chinese martial arts, Taekwondo, and gymnastics). Sample 1 included 213 athletes (118 females and 95 males, *M*_age_ = 18.93, *SD* = 3.88) for exploratory factor analysis, while Sample 2 included 213 people (111 females and 102 males, *M*_age_ = 19.05, *SD* = 3.81) for confirmatory factor analysis. Both samples were combined to calculate internal consistency coefficients. For retest reliability, 143 athletes participated in a 1-week retest (Sample 3: 64 females and 79 males, *M*_age_ = 19.62, *SD* = 3.92), and 119 athletes participated in a 2-week retest (Sample 4: 51 females and 68 males, *M*_age_ = 19.17, *SD* = 3.78). In addition, 239 athletes completed the AIS-NCA, and the scales used to examine validity (Sample 5: 148 females and 91 males, *M*_age_ = 18.87, *SD* = 3.84).

Students were randomly divided into two parallel samples based on gender and class. These two samples were used to conduct exploratory factor analysis (Sample 6: 389 students in total, 191 females and 198 males, *M*_age_ = 15.79, *SD* = 1.05) and confirmatory factor analysis (Sample 7: 390 students in total, 177 females and 213 males, *M*_age_ = 15.77, *SD* = 0.71), respectively. These two samples were also combined for the calculation of internal consistency coefficients. 202 students participated a retests after 2-week intervals and completed the scales used to test the validity of the AIS-NCA at the time of retesting (Sample 8: 97 females and 105 males, *M*_age_ = 15.33, *SD* = 0.48).

The study received ethical approval from the Ethics Committee of the Shanghai Research Institute of Sports Science (Shanghai Anti-doping Agency; No. LLSC20220005), and adheres to the ethical principles outlined in the Declaration of Helsinki and the Standards for Ethics in Sport and Exercise Science Research (Tyebkhan, 2003; Harriss et al., 2019). Prior to the test, all participants provided written consent. Additionally, athletes and students obtained permission from their respective sports teams or schools before participating in the study.

### Measures

#### Athens insomnia scale for non-clinical application

The Athens insomnia scale for non-clinical application (AIS-NCA) was adapted from the Athens Insomnia Scale and comprises seven items, each rated on a 5-point scale. The options for each item are represented by text that matches the content of the item. The scale comprises two dimensions, sleep problems and daytime functioning, which allow for the computation of two subscale scores and a total scale score. Higher scores indicate more severe sleep quality issues (Sattler et al., 2021).

In this study, the Chinese version of the AIS-NCA was developed through a rigorous process, which included translation and back-translation, after getting permission from the authors of AIS-NCA. Specifically, two sports science researchers and one teacher, all with professional backgrounds in psychology, independently translated the original scale into Chinese. These translations were then compiled and revised by a sports science researcher with a psychology background and a specialist with backgrounds in both English and kinesiology after a thorough discussion. The revised version was then pilot-tested multiple times with athletes of varying sports and age groups, and the text was further revised based on their feedback. The Chinese version of the scale for back-translation was then formed.

Then, the initial version was back-translated into English by a psychological specialist who obtained a psychology degree from an English-speaking country but did not take part in the initial translation. A native English-speaking professional then reviewed the items and suggested changes until there was a consensus on the semantics. The Chinese version of the scale obtained through this process was used in this study.

#### Pittsburgh sleep quality index

Pittsburgh sleep quality index (PSQI) is a widely used sleep scale that has gained popularity in the field of sports science. It consists of 19 self-rated and five other-rated items, with only 18 self-rated items being scored. These items assess sleep quality, sleep duration, sleep efficiency, sleep disturbance, sleep medication, and daytime dysfunction and require responses based on the previous month’s state (Buysse, 2014). In this study, the scale was employed for analyzing convergent validity. To provide a more detailed characterization of sleep and following previous studies, separate scores were calculated for different aspects of sleep in addition to the total PSQI score (Bender et al., 2018a,b; Cellini et al., 2021; Driller et al., 2022). These dimensions included subjectively evaluated sleep quality, total sleep time, sleep latency, calculated sleep efficiency, total sleep disturbances score, and total daytime dysfunction score (a higher computed sleep efficiency score indicates better sleep quality). Sleep medication was not analyzed separately due to the restrictions on medication use in athletes and students. In this study, the overall internal consistency coefficients for the PSQI were Cronbach’s α _athlete_ = 0.719 and Cronbach’s α _student_ = 0.689.

#### Epworth sleepiness scale

Epworth sleepiness scale (ESS) is a widely used scale for assessing daytime sleepiness. It consists of eight daily situations in which respondents rate their likelihood of falling asleep on a four-point scale, with higher scores indicating higher levels of daytime sleepiness (Johns, 1991). In this study, the scale was employed for analyzing convergent validity. High school students did not complete the ESS because school administrators deemed some items inappropriate for completion by underage high school students. Athletes were asked to respond based on their status in the previous month in this study, and the internal consistency coefficient for the ESS was Cronbach’s α =0.796.

#### Athlete sleep screening questionnaire

Athlete sleep screening questionnaire (ASSQ) is a scale developed for athletes to screen and guide interventions for sleep problems (Samuels et al., 2016). It consists of 16 items that are divided into three categories: Sleep Difficulty Score (SDS), which is the core of the ASSQ and consists of 5 items, with higher scores indicating more significant sleep difficulties; Modifiers that consist of three indexes: sleep breathing disorder, travel, and sleep type; and other items related to napping, caffeine, and electronic devices, respectively, that cannot be classified into SDS but can provide direct suggestions for sleep improvement strategies. In this study, only the SDS was used to represent the sleep quality of the athletes based on the original scale asking athletes to report based on their status in the most recent period. In this study, the SDS was employed for analyzing convergent validity. The internal consistency coefficient for the SDS in this study was Cronbach’s α = 0.423.

#### Warwick-Edinburgh mental well-being scale

The Warwick-Edinburgh Mental Well-Being Scale (WEMWBS) consists of 14 items and reflects mental well-being (Tennant et al., 2007). Participants were instructed to rate their experiences on a 5-point scale ranging from “never” to “always.” This single-dimensional scale yields higher scores for higher levels of mental well-being. The Chinese version of this scale has been widely applied and has demonstrated good reliability and validity across various populations in China. Drawing from previous research (Sattler et al., 2021), this study employs this measurement of well-being to analyze the discriminant validity among athletes. Additionally, it is hypothesized that a significant negative correlation will be evident between the scores of WEMWBS and AIS-NCA (Tang et al., 2017). In this study, the internal consistency coefficient of WEMWBS among athletes was Cronbach’s α = 0.951.

#### General self-efficacy scale

The General Self-Efficacy Scale (GSES) consists of 10 items and reflects the level of optimistic self-beliefs. Participants rate themselves on a 4-point scale according to their subjective experiences, with higher scores indicating higher levels of self-efficacy. The Chinese version of this scale is widely used and demonstrates good reliability and validity (Zhang and Schwarzer, 1995). The assessment of students was integrated into an internal psychological evaluation within the school, where the GSES had already been included. However, due to administrative considerations and caution regarding introducing a scale related to mental health, the timely incorporation of WEMWBS into this assessment was not achieved. Research has indicated that general self-efficacy positively predicts sleep quality (Ghose et al., 2023). Thus, in this study, it can be used for analyzing the discriminant validity among students as an alternative. The same hypothesis of a significant negative correlation between the total score and individual dimensions applies. In this study, the internal consistency coefficient of GSES among students was Cronbach’s α = 0.911.

### Procedure

Athletes were recruited to participate in this study by sports teams in both Sample 1 and Sample 2. The AIS-NCA was completed online by all athletes. Depending on the sports team’s schedule, most athletes completed it independently in the designated testing room; for sports teams that could not schedule it, the teams themselves arranged the accomplishment of the scale. Athletes who had no significant competitions in the following month were recruited to complete AIS-NCAs twice at 1-or 2-week intervals, respectively, for Samples 3 and 4, and all retests were completed online. Athletes were recruited for Sample 5 to complete the AIS-NCA, PSQI, ESS, and ASSQ during long-term closed training sessions at the training base, and all scales were completed online.

Following approval from school administrators, the school psychologist organized the students to complete the AIS-NCA according to the class schedule. Those who were able to complete the AIS-NCA in class on the designated date in the course schedule used the paper-based scale; for those who were unable to coordinate their time, the class teacher distributed the test link, and the students completed the scale online after school on their own (Sample 6 and Sample 7). A retest was organized in those classes that were willing to participate in the retest after a 2-week interval, and students who participated completed the PSQI at the same time (Sample 8), all using the paper-based questionnaire.

### Statistical analysis

Data were organized using Microsoft Excel 2016. SPSS 26.0 was used for exploratory factor analysis (EFA) and reliability and validity tests, and Amos 20.0 was used for confirmatory factor analysis (CFA). The Kaiser-Meyer-Olkin and Bartlett’s spherical tests were used in the EFA to determine whether the data were suitable for factor analysis. Principal component analysis with maximum variance rotation was used to analyze the factors with an eigenvalue greater than 1. Items with factor loadings of at least 0.320 were retained (Costello and Osborne, 2005). Based on prior research, items with multiple high loadings were removed using two criteria. First, items with loadings differing by less than 0.150 across different factors were deleted (Worthington and Whittaker, 2006; Sattler et al., 2021). Second, items with strong loadings exceeding 0.500 on more than one factor were also removed (Costello and Osborne, 2005).

In the CFA, model fit was evaluated using the χ^2^/*df* ratio, comparative fit index (CFI), adjusted goodness of fit index (AGFI), Tucker–Lewis index (TLI), and root mean square error of approximation (RMSEA). Previous studies were consulted to establish the acceptable criteria: a χ^2^/*df* ratio below 5.00 (preferably below 2), CFI, AGFI, and TLI values greater than 0.90, and RMSEA values below 0.08 (Nock et al., 2009; Sattler et al., 2021). Furthermore, multiple-group analysis (multiple-group CFA) was conducted to examine the measurement invariance across different genders of athletes and students. Additionally, comparisons were also carried out between athlete and student samples. Various models within the multiple-group CFA were systematically compared, including the Unconstrained Model, Measurement Weights Model, Structural Covariances Model, and Measurement Residuals Model.

The intraclass correlation coefficient (ICC) was utilized to examine test–retest reliability. Convergent and discriminant validity were evaluated using Pearson correlation coefficients. Internal consistency reliability was assessed using both Cronbach’s α coefficient and McDonald’s ω coefficient.

## Results

### Athletes

#### EFA

An exploratory factor analysis was conducted on the Chinese version of the AIS-NCA using Sample 1. The results of Kaiser Meyer Olkin test and Bartlett’s test of sphericity showed that the sample was sufficient for factor analysis [KMO = 0.783; χ^2^(21) = 467.817, *p* < 0.001]. The results revealed two factors with eigenvalues greater than 1 (Factor 1 = 3.187, Factor 2 = 1.274), which accounted for 63.73% of the variance (Factor 1 = 33.35%, Factor 2 = 30.38%). Item 4 (“The overall quality of my sleep was usually…”) had high and similar loadings on both factors and met the criteria for exclusion.

After the removal of item 4, the factor analysis was rerun, and revealed that KMO = 0.707, and the result of Bartlett’s spherical test also indicated suitability for factor analysis [χ^2^(15) = 330.222, *p* < 0.001]. The analysis revealed two factors with eigenvalues greater than 1 (Factor 1 = 2.644, Factor 2 = 1.274), accounting for 65.08% of the variance (Factor 1 = 35.53%, Factor 2 = 29.55%). The remaining six items on the two factors were consistent with the original scale (Table 1), and the two factors were named Sleep Problems and Daytime Functioning, based on the nomenclature of the original scale. Subsequent analyses were conducted with the 6-item AIS-NCA among athletes.

**Table 1:** Factor loadings of the AIS-NCA among athletes.

	7 items		6 items	
	Sleep problems	Daytime functioning	Sleep problems	Daytime functioning
Item 1	**0.580**	0.350	**0.541**	0.361
Item 2	**0.843**	0.075	**0.862**	0.104
Item 3	**0.779**	0.020	**0.815**	0.050
Item 4	**0.624**	**0.512**	-	-
Item 5	0.260	**0.814**	0.238	**0.822**
Item 6	0.099	**0.842**	0.101	**0.855**
Item 7	0.072	**0.756**	0.078	**0.763**

The bold numbers in the table indicate that the item can be attributed to that dimension.

#### CFA

**Figure 1:**
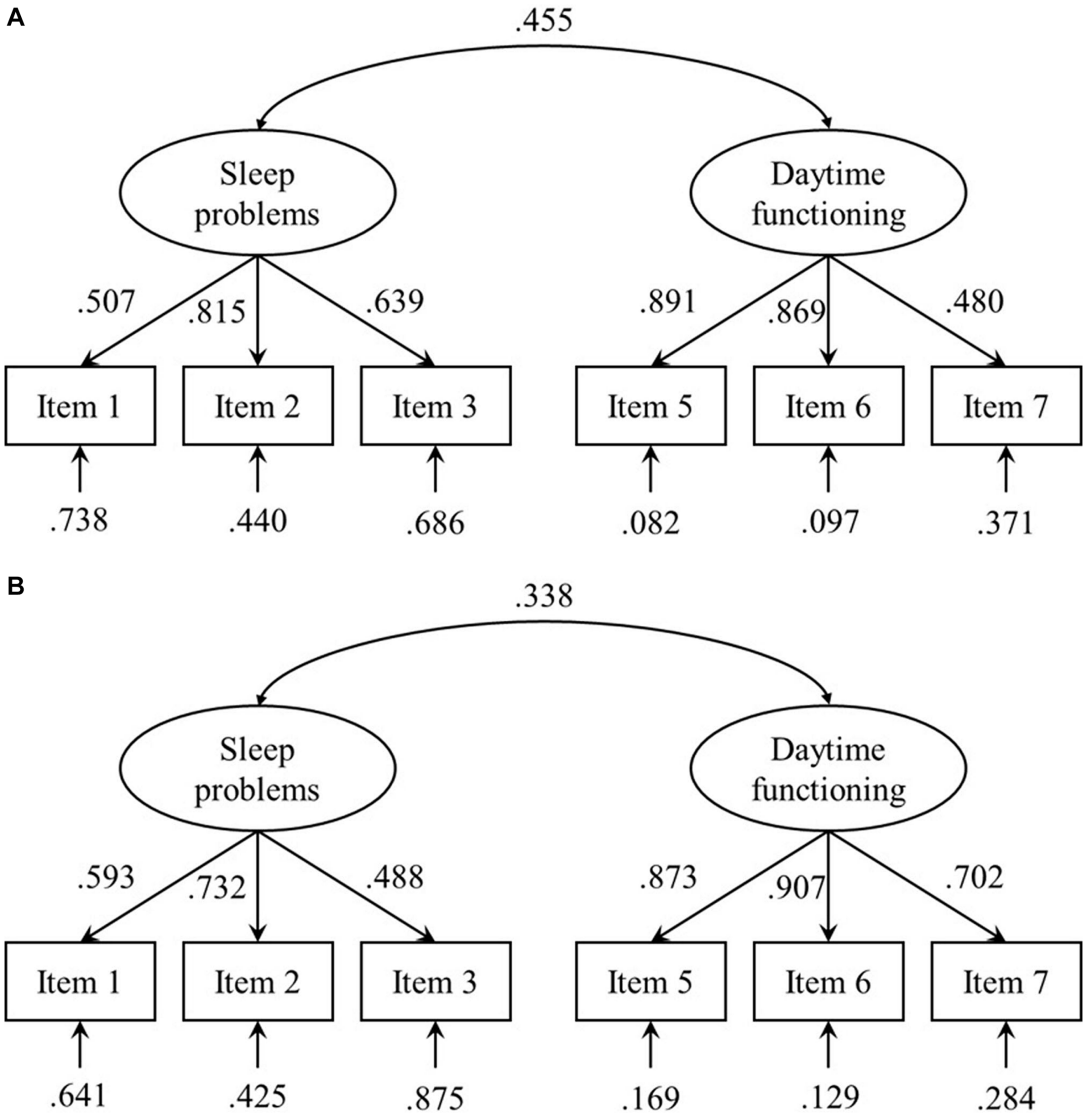
CFA parameter estimates for athletes **(A)** and students **(B)**.

CFA was initially conducted on a one-factor model using six items, leading to χ^2^/*df* = 9.490, CFI = 0.803, AGFI = 0.725, TLI = 0.671, and RMSEA = 0.200, indicating a poor fit. Subsequently, a CFA was carried out on a two-factor structure derived from exploratory factor analysis, leading to χ^2^/*df* = 2.217, CFI = 0.975, AGFI = 0.929, TLI = 0.953, and RMSEA = 0.076, demonstrating a good model fit (refer to Figure 1A for each path loading).

#### Invariance analysis

Expanding on the foundation of the CFA, an additional analysis was undertaken using Sample 2 to explore the measurement invariance of the AIS-NCA across athletes of different genders through a multi-group analysis. The constrained measurement weights model, where loadings were constrained to be equal, was compared to the Unconstrained Model. The results showed a negligible difference with Δχ^2^ = 1.732, Δ*df* = 4, and *p* = 0.785. Additionally, the changes in fit indices were minimal, with ΔCFI = 0.006, ΔAGFI = 0.017, ΔTLI = 0.022, and ΔRMSEA = −0.012 when compared to the Unconstrained Model. Furthermore, a structural covariances model was contrasted with the Measurement Weights Model. The comparison indicated a difference with Δχ^2^ = 14.297, Δ*df* = 3, and *p* = 0.003. The differences in fit indices were observed, including ΔCFI = −0.032, ΔAGFI = −0.024, ΔTLI = −0.037, and ΔRMSEA = 0.018 when compared to the Measurement Weights Model. Lastly, a measurement residuals model was compared to the Structural Covariances Model. The results revealed no statistically significant difference with Δχ^2^ = 6.783, Δ*df* = 6, and *p* = 0.341. The changes in fit indices were negligible, with ΔCFI = −0.002, ΔAGFI = 0.009, ΔTLI = 0.014, and ΔRMSEA = −0.006 when compared to the Structural Covariances Model.

#### Reliability

The one-week retest intraclass correlation coefficients (ICC) were significant and indicative of good reliability for the total score (ICC = 0.737, *p* < 0.001), the sleep problems dimension (ICC = 0.745, *p* < 0.001), and the daytime functioning dimension (ICC = 0.599, *p* < 0.001). The two-week retest ICC was also significant but slightly lower for the total score (ICC = 0.517, *p* < 0.001), the sleep problems dimension (ICC = 0.525, *p* < 0.001), and the daytime functioning dimension (ICC = 0.451, *p* < 0.001).

The internal consistency coefficients were also good, with Cronbach’s α = 0.729 and McDonald’s ω = 0.708 for the six AIS-NCA items, Cronbach’s α = 0.666 and McDonald’s ω = 0.721 for the sleep problems, and Cronbach’s α = 0.773 and McDonald’s ω = 0.784 for the daytime functioning.

#### Convergent and discriminant validity

With the exception of the non-significant correlation coefficient between the AIS-NCA sleep problem scores and PSQI-sleep duration, there were significant correlations observed between the total AIS-NCA scores and the scores of both dimensions with the total PSQI scores and scores of subscales, as well as with ESS and ASSQ-SDS, indicating strong convergent validity. Moreover, moderate negative correlations were observed between AIS-NCA total scores, its dimension scores, and WEMWBS, demonstrating good discriminant validity (refer to Table 2 for correlation coefficients).

**Table 2:** Correlation coefficients between AIS-NCA, PSQI, ESS, ASSQ, and WEMWBS (Athletes).

	n	AIS-NCA Total Score	AIS-NCA Sleep problems	AIS-NCA Daytime functioning
PSQI	232	0.637 (*p* < 0.001)	0.529 (*p* < 0.001)	0.596 (*p* < 0.001)
- Subjective sleep quality	232	0.620 (*p* < 0.001)	0.554 (*p* < 0.001)	0.527 (*p* < 0.001)
- Sleep latency	232	0.530 (*p* < 0.001)	0.495 (*p* < 0.001)	0.422 (*p* < 0.001)
- Sleep duration	232	0.149 (*p* = 0.023)	0.079 (*p* = 0.228)	0.199 (*p* = 0.002)
- Sleep efficiency	232	−0.313 (*p* < 0.001)	−0.204 (*p* = 0.002)	−0.365 (*p* < 0.001)
- Sleep disturbance	232	0.554 (*p* < 0.001)	0.536 (*p* < 0.001)	0.417 (*p* < 0.001)
- Daytime functioning	232	0.533 (*p* < 0.001)	0.345 (*p* < 0.001)	0.628 (*p* < 0.001)
ESS	239	0.637 (*p* < 0.001)	0.529 (*p* = 0.001)	0.596 (*p* < 0.001)
ASSQ-SDS	239	0.299 (*p* < 0.001)	0.213 (*p* < 0.001)	0.329 (*p* < 0.001)
WEMWBS	239	−0.424 (*p* < 0.001)	−0.440 (*p* < 0.001)	−0.442 (*p* < 0.001)

### Students

#### EFA

An exploratory factor analysis was conducted on the Chinese version of the AIS-NCA using Sample 6. The results of Kaiser Meyer Olkin test and Bartlett’s test of sphericity showed that the sample was sufficient for factor analysis [KMO = 0.799; χ^2^(21) = 1045.499, *p* < 0.001]. The results revealed two factors with eigenvalues greater than 1 (Factor 1 = 3.359, Factor 2 = 1.204), which accounted for 65.18% of the variance (Factor 1 = 37.74%, Factor 2 = 27.44%). Similar to the athlete sample, item 4 had high and similar loadings on both factors and met the criteria for exclusion.

After the removal of item 4, the factor analysis was rerun and revealed that KMO = 0.743, and the result of Bartlett’s spherical test also indicated suitability for factor analysis [χ^2^(15) = 773.024, *p* < 0.001]. The analysis revealed two factors with eigenvalues greater than 1 (Factor 1 = 2.810, Factor 2 = 1.163), accounting for 66.22% of the variance (Factor 1 = 40.30%, Factor 2 = 25.92%). The remaining six items on the two factors were consistent with the original scale (Table 3), and the two factors were named Sleep Problems and Daytime Functioning, based on the nomenclature of the original scale. Subsequent analyses were conducted with the 6-item AIS-NCA among students.

**Table 3:** Factor loadings of the AIS-NCA among students.

	7 items		6 items	
	Sleep problems	Daytime functioning	Sleep problems	Daytime functioning
Item 1	**0.617**	0.308	**0.575**	0.331
Item 2	**0.759**	0.183	**0.757**	0.208
Item 3	**0.697**	−0.064	**0.765**	−0.044
Item 4	**0.639**	**0.527**	-	-
Item 5	0.172	**0.872**	0.158	**0.879**
Item 6	0.157	**0.899**	0.157	**0.906**
Item 7	0.127	**0.815**	0.130	**0.819**

The bold numbers in the table indicate that the item can be attributed to that dimension.

#### CFA

CFA was initially conducted on a one-factor model using six items, leading to χ^2^/*df* = 15.062, CFI = 0.840, AGFI = 0.747, TLI = 0.733, and RMSEA = 0.190, indicating a poor fit. Subsequently, a CFA was carried out on a two-factor structure derived from exploratory factor analysis, leading to χ^2^/*df* = 3.037, CFI = 0.979, AGFI = 0.947, TLI = 0.961, and RMSEA = 0.072, demonstrating a good model fit (refer to Figure 1B for each path loading).

#### Invariance analysis

An analysis of measurement invariance among students of different genders was conducted using Sample 7. The constrained measurement weights model was compared to the Unconstrained Model. The results showed a slight difference with Δχ^2^ = 10.388, Δ*df* = 4, *p* = 0.034. Moreover, there were negligible changes in the fit indices, including ΔCFI = −0.009, ΔAGFI = 0, ΔTLI = −0.002, and ΔRMSEA = 0.002, in comparison to the Unconstrained Model. Additionally, the structural covariances model was contrasted with the Measurement Weights Model. The comparison did not find a difference with Δχ^2^ = 3.351, Δ*df* = 3, *p* = 0.341. The variations in fit indices were minimal, with ΔCFI = 0, ΔAGFI = 0.005, ΔTLI = 0.006, and ΔRMSEA = −0.004, compared to the Measurement Weights Model. Moreover, the measurement residuals model was compared to the Structural Covariances Model. The results showed no difference with Δχ^2^ = 6.082, Δ*df* = 6, *p* = 0.414. The changes in fit indices were also negligible, with ΔCFI = 0, ΔAGFI = 0.008, ΔTLI = 0.009, and ΔRMSEA = −0.006, in comparison to the Structural Covariances Model.

Furthermore, an invariance analysis was conducted between athletes and students using Samples 2 and 7. The constrained measurement weights model, compared to the Unconstrained Model, yielded Δχ^2^ = 5.773, Δ*df* = 4, *p* = 0.217. Additionally, the changes in fit indices were minor, with ΔCFI = −0.002, ΔAGFI = 0.004, ΔTLI = 0.007, and ΔRMSEA = −0.004, when compared to the Unconstrained Model. Likewise, the structural covariances model was compared with the Measurement Weights Model. The comparison revealed a significant difference with Δχ^2^ = 31.397, Δ*df* = 3, and *p* < 0.001. The differences in fit indices were minor, including ΔCFI = −0.024, ΔAGFI = −0.019, ΔTLI = −0.027, and ΔRMSEA = 0.016, when compared to the Measurement Weights Model. Lastly, the measurement residuals model was contrasted with the Structural Covariances Model. The results demonstrated a statistically significant difference with Δχ^2^ = 29.176, Δ*df* = 6, and *p* < 0.001. The changes in fit indices were minor, with ΔCFI = −0.020, ΔAGFI = −0.004, ΔTLI = −0.008, and ΔRMSEA = 0.003, compared to the Structural Covariances Model.

#### Reliability

The two-week retest ICC was also significant but slightly lower for the total score (ICC = 0.649, *p* < 0.001), the sleep problems dimension (ICC = 0.639, *p* < 0.001), and the daytime functioning dimension (ICC = 0.576, *p* < 0.001).

The internal consistency coefficients were also good, with Cronbach’s α = 0.729 and McDonald’s ω = 0.701 for the six AIS-NCA items, Cronbach’s α = 0.575 and McDonald’s ω = 0.593 for the sleep problems, and Cronbach’s α = 0.865 and McDonald’s ω = 0.875 for the daytime functioning.

#### Convergent and discriminant validity

Significant correlations were observed between the total AIS-NCA scores and the scores of both dimensions with the total PSQI scores and subscales scores, indicating strong convergent validity. Moreover, moderate negative correlations were observed between AIS-NCA total scores, its dimension scores, and GSES, demonstrating good discriminant validity (refer to Table 4 for correlation coefficients).

**Table 4:** Correlation coefficients between AIS-NCA, PSQI, and GSES (Students).

	*n*	AIS-NCA total score	AIS-NCA sleep problems	AIS-NCA daytime functioning
PSQI	188	0.772 (*p* < 0.001)	0.612 (*p* < 0.001)	0.611 (*p* < 0.001)
- Subjective sleep quality	188	0.692 (*p* < 0.001)	0.616 (*p* < 0.001)	0.476 (*p* < 0.001)
- Sleep latency	188	0.563 (*p* < 0.001)	0.651 (*p* < 0.001)	0.233 (*p* = 0.001)
- Sleep duration	188	0.296 (*p* < 0.001)	0.144 (*p* = 0.049)	0.329 (*p* < 0.001)
- Sleep efficiency	188	−0.326 (*p* < 0.001)	−0.258 (*p* < 0.001)	−0.257 (*p* < 0.001)
- Sleep disturbance	188	0.591 (*p* < 0.001)	0.573 (*p* < 0.001)	0.359 (*p* < 0.001)
- Daytime functioning	188	0.525 (*p* < 0.001)	0.146 (*p* = 0.046)	0.695 (*p* < 0.001)
GSES	195	−0.389 (*p* < 0.001)	−0.165 (*p* = 0.021)	−0.457 (*p* < 0.001)

## Discussion

Athletes’ sleep is essential for more than simply health reasons; it also helps them perform better in competition (Dorrian and Dinges, 2006; Malhotra, 2017; Bonnar et al., 2018; O’Donnell et al., 2018; Walsh et al., 2021). Accurate and reliable quantitative assessments of their sleep are required to boost athletes’ health and performance by describing, detecting, and improving their sleep.

Scales have long been a popular measure for monitoring sleep quality. In practice, classical sleep quality assessment scales and screening tools have contributed significantly to research on sleep quality risk assessment and enhancement in athletes (Mah et al., 2018; Bender et al., 2018a,b; Halson, 2019; Rebello et al., 2022; Suppiah et al., 2022), facilitating the development and application of sleep science in sports. Yet, researchers have grown increasingly conscious that the scales used to assess sleep quality in athletes were designed largely for clinical use, despite the fact that the vast majority of populations tested did not include patients with clinical issues. Consequently, using scales intended for clinical purposes may lead to potential deficiencies or problems in the outcomes and practice of the study (Sattler et al., 2021).

To address concerns about potential scale challenges, researchers created the AIS-NCA and demonstrated its validity and applicability in the general population (Sattler et al., 2021). This study aimed to revise and validate the AIS-NCA scale among Chinese athletes and further investigate the potential influence of cultural differences or professional characteristics in a non-athlete population. This research provided an initial exploration of the applicability of AIS-NCA among Chinese athletes. The findings demonstrated that AIS-NCA can be effectively utilized within the Chinese athlete population. However, the revised scale exhibited both similarities and notable differences compared to the original version. Regarding the scale’s dimensions and items, the Chinese version of the AIS-NCA covers the same two categories as the original scale: sleep problems and daytime functioning. This study also showed that the structure is not exclusive to athletes. Although the authors of the AIS did not explicitly propose this two-factor structure, they did suggest, at an operational level, that the items belonging to the first dimension be used separately as short scales (Soldatos et al., 2000). This suggestion indirectly supports the two-factor structure. Perhaps because the AIS was developed based on the ICD-10, clinical use as a diagnostic aid is prioritized over differentiating the dimensions of sleep quality (Soldatos et al., 2000; Hallit et al., 2019). Nonetheless, for non-clinical purposes, the division into two dimensions allows for a more comprehensive analysis and investigation of the links between aspects of sleep quality (Sattler et al., 2021). It might also present new approaches to investigating the relationship between sleep and sports performance.

The Chinese version of AIS-NCA demonstrates consistency with the original questionnaire in terms of dimensions, but a notable difference appears in one specific item. Whether among athletes or non-athletes, the items related to subjective sleep quality or satisfaction with sleep quality exhibit strong and similar loadings on both dimensions. It can be inferred that Chinese athletes’ cognition of subjective sleep quality may differ from that of the groups involved in previous studies, which may be the main reason for this phenomenon.

Some researchers have emphasized that sleep quality can be defined as an individual’s self-satisfaction with various aspects of their sleep experience and is a concept with ambiguous boundaries (Harvey et al., 2008; Nelson et al., 2022). Studies using interviews and other methods have revealed that when discussing sleep quality, both insomnia patients and the general population include not only sleep-related issues such as nighttime awakenings but also experiences associated with daytime functioning, such as fatigue and recovery (Harvey et al., 2008). Additionally, other researchers have suggested that differences in how individuals define ideal sleep can influence their subjective perceptions (Buysse, 2014), indicating that subjective sleep quality may be linked to the content of sleep quality definitions.

Considering that this phenomenon was observed among athletes and non-athletes in this study, it is conceivable that this perception of sleep quality may stem from cultural factors. For instance, researchers found that objective sleep quality in Japanese university students was lower than that of European-Canadian students, but they did not report matching levels of fatigue and health. However, Asian-Canadians living in a Canadian cultural context showed patterns similar to European-Canadians (Cheung et al., 2021). While cross-cultural research on this topic is somewhat limited, existing studies suggest that cultural norms surrounding sleep indeed exist and may influence subjective experiences, potentially relating to the content of sleep quality.

Since cultural factors may account for the differences in AIS-NCA items, one might wonder whether these differences could also be associated with the athlete population. Examining the structural disparities of AIS-NCA between athletes and non-athletes, it becomes evident that both groups exhibit consistent patterns, not only regarding the phenomenon related to subjective sleep quality items but also in the loadings of all the items. These disparities are only evident when introducing covariance and residuals into the model. Based on this finding, it is reasonable to infer that AIS-NCA demonstrates a comparable structure among athletes and non-athletes, with item differences more likely stemming from cultural influences rather than occupational factors.

Regarding the measurement invariance of the AIS-NCA, this study revealed a high level of consistency in structure and loadings among athletes of different genders and between athlete and student samples. However, some limitations emerged when incorporating controlled covariances and residual models. In the initial research introducing the AIS-NCA, scholars demonstrated good scalar invariance across gender, age groups, sleep disorders, and cross-linguistic samples in a large-scale study (Sattler et al., 2021). In this study, different gender samples exhibited satisfactory equivalence at the structural and loading levels, yet slightly weaker equivalence after introducing residuals, compared to previous research. A similar phenomenon was observed in the model comparison between athletes and the general population. This could be related to the exclusion of the subjective sleep quality item in this study and potential underlying cultural differences to some extent. This outcome might suggest that when applying the AIS-NCA to Chinese athletes and the general population, more attention to the issue of invariance is required, especially compared to its application in English-or German-speaking contexts. Nevertheless, research in this area remains limited, highlighting the need for further analysis and interpretation of cross-population measurement invariance in a broader array of populations.

Regarding the validity of the AIS-NCA, both for athletes and the non-athlete population, significant positive correlations were found between PSQI, ESS, ASSQ, and AIS-NCA scores, indicating strong Convergent Validity. AIS-NCA’s Discriminant Validity also demonstrated the expected patterns among athletes and the general population. Overall, the revised AIS-NCA exhibited robust validity. Additionally, it is noteworthy that the analysis of Convergent Validity involved several indicators. These findings not only support the validity of the AIS-NCA but also shed light on its unique characteristics regarding the assessment of various sleep features. Such insights contribute positively to understanding the features of the AIS-NCA tool and its application recommendations.

Concerning the relationship between AIS-NCA and PSQI, specifically focusing on the sleep disorders and daytime functioning dimensions of PSQI, noticeable differences in correlation coefficients of the two dimensions of AIS-NCA were observed. This outcome further underscores the relatively strong validity of AIS-NCA’s two dimensions. However, the correlation coefficients were relatively low for AIS-NCA total scores or the scores of its two dimensions with sleep duration and sleep efficiency in PSQI. This result indicates that AIS-NCA itself may not fully describe the intricacies of sleep characteristics and should be complemented by consensus sleep diaries or other objective methods (Carney et al., 2012).

In contrast to the findings for the daytime functioning dimension in the PSQI, the AIS-NCA scores displayed moderate correlations with the score for both dimensions on the Epworth Sleepiness Scale (ESS). However, the differences in correlation coefficients between the sleep disturbance and daytime functioning dimensions were not as pronounced as those observed on the PSQI. This phenomenon is consistent with the results obtained in the original questionnaire (Sattler et al., 2021). Two possible explanations for these findings are proposed. Firstly, the ESS primarily reflects sleepiness rather than overall daytime functioning (Johns, 1991). While sleepiness requires recuperation through sleep (Mairesse et al., 2017), it does not necessarily represent daytime functioning in its entirety. Secondly, some ESS items may be unfamiliar or difficult to imagine for Chinese athletes, such as “watching TV” and “reading, “which may not be everyday activities in their daily lives. As a result, the validity of the ESS may be reduced due to reliance on imagination.

The correlation coefficients obtained on the SDS were low. This result may be attributable to the ASSQ’s intended use, which includes assessing athletes for sleep issues and making hierarchical intervention recommendations (Samuels et al., 2016). The SDS employs a multiple-choice format, and the ability to provide a precise evaluation of overall or specific aspects of sleep quality in athletes is not deemed critical. Looking at this more constructively, augmenting the use of the ASSQ with the AIS-NCA in athletes may have added advantages. Specifically, incorporating AIS-NCA in the periodic assessment of athletes without sleep disorders could enhance training and recovery quality, while tracking athletes with sleep disorders could facilitate the evaluation of intervention efficacy and progress.

Regarding reliability, the overall internal consistency coefficient is satisfactory, and the results obtained from athletes are similar to those of the original study. However, it was observed that non-athlete students’ consistency coefficient for sleep problems is not high enough. It is essential to highlight that even among athletes, the consistency coefficient for this dimension is also relatively low. This observation is reflected in the loadings of the factors. This discrepancy may be attributed to the complex relationship between sleep indicators, as even the PSQI questionnaire lacks conclusive evidence regarding the number and content of dimensions (Raniti et al., 2018; Jia et al., 2019; Guo, 2022). Therefore, further investigation of the relationship between different sleep indicators may be warranted, possibly incorporating additional moderator variables.

The retest reliability of the AIC-NCA was evaluated over 1-week and 2-week intervals among athletes and non-athletes, respectively. The reliability of the 1-week retest was good and only slightly lower than that reported in previous studies (Sattler et al., 2021). Although still satisfactory, the 2-week retest reliability was lower than the 1-week retest. This disparity may be attributed to factors such as training and fatigue, which can affect athletes’ sleep quality (Lastella et al., 2018, 2020). This could also be true for students who represented the general population and were subject to factors such as on-campus activities and exams, which could alter their sleep status during retest. It is suggested that the sleep quality obtained from AIC-NCA may reflect a more recent period. Thus, it requires consideration of recent training and recovery to avoid misinterpretation when athletes are asked to report on a more extended period. However, it also suggests that AIC-NCA may be more sensitive to changes in sleep quality and suitable for the evaluation and timely intervention of athletes’ sleep quality over shorter or specific periods, such as different phases of the season (Biggins et al., 2021; Saidi et al., 2022).

Overall, this study proves that the Chinese version of AIS-NCA is effective for assessing sleep among Chinese athletes, demonstrating good reliability and validity within a short timeframe (1 month). It comprehensively evaluates athletes’ sleep by considering sleep problems and daytime functioning. When using AIS-NCA with Chinese athletes, the 6-item version is recommended. However, considering potential cultural factors and facilitating cross-cultural research or subjective sleep quality analyses, completion of the 7-item version is suggested while excluding the excluded item from calculations. Moreover, the Chinese version of AIS-NCA also exhibits promising applicability among high school students outside the athlete population, which carries positive implications for cross-population comparisons, especially in student-athlete research. Nevertheless, caution is advised when applying it to high school students based on the validity obtained in this study.

The Chinese version of AIS-NCA complements existing methods for assessing athlete sleep quality, providing valuable support for research and applications related to sports training, fatigue recovery, and athlete mental health. However, this study has several limitations. Firstly, it represents a preliminary revision of the Chinese version of AIS-NCA, lacking sensitivity testing concerning athletes’ training and competitions, and assessments across different time frames, which leaves the full potential of its application value and characteristics in sports training unexplored. Secondly, the criterion measures used were self-report scales, making the response subjective, and objective measures were not employed due to practical constraints. Thirdly, the non-athlete group was confined to high school students with similar time management in their daily routines, restricting the explanatory power regarding cultural and occupational influences and the generalizability of use in a broader population.

Future research can further integrate daily training and actual competitions to examine the reliability of AIS-NCA in assessing sleep quality during these activities within shorter timeframes, and explore ways to incorporate it into training programs. Additionally, more profound explorations of the significance of the two dimensions should be conducted by combining objective sleep indicators. Moreover, it is imperative to consider cultural differences and the impact of occupation and population characteristics to delve into the distinctive features of Chinese cultural cognition regarding sleep quality. Furthermore, the effectiveness and invariance of AIS-NCA should be further tested in a broader population, with particular attention to the role of population characteristics. This will expand the scale’s applicability in Chinese people and provide insights into analyzing sleep characteristics among Chinese athletes.

## Conclusion

The Chinese version of the Athens Insomnia Scale for Non-clinical Application retains the exact two dimensions as the original scale: sleep problems and daytime functioning, while excluding one item related to subjective sleep quality. The Chinese version of the scale demonstrates good reliability and validity, making it suitable for assessing sleep quality among Chinese athletes. The structure among Chinese athletes may reflect specific cultural differences. While it shows promise for application in other Chinese populations, a caution approach is recommended.

## Data availability statement

The raw data supporting the conclusions of this article will be made available by the authors, without undue reservation.

## Ethics statement

The studies involving humans were approved by the Ethics Committee of Shanghai Research Institute of Sports Science (Shanghai Anti-doping Agency). The studies were conducted in accordance with the local legislation and institutional requirements. Written informed consent for participation in this study was provided by the participants. For minors, written informed consent was provided by their legal guardians or next of kin.

## Author contributions

CT and JQ conceptualized the study. CT, JW, GC, CC, JY, and JL organized the data collection. CT wrote the first draft of the manuscript. CT and JW run the analyses and wrote the results section. All authors contributed to the article and approved the submitted version.
